# Maize plant expresses SWEET transporters differently when interacting with *Trichoderma asperellum* and *Fusarium verticillioides*, two fungi with different lifestyles

**DOI:** 10.3389/fpls.2023.1253741

**Published:** 2023-09-27

**Authors:** Montserrat López-Coria, Fernando Guzmán-Chávez, Roberto Carvente-García, Daniela Muñoz-Chapul, Tomás Sánchez-Sánchez, Juan Manuel Arciniega-Ruíz, Beatriz King-Díaz, Sobeida Sánchez-Nieto

**Affiliations:** Dpto. de Bioquímica, Facultad de Química, Conjunto E. Universidad Nacional Autónoma de México, Mexico City, Mexico

**Keywords:** *Zea mays*, *Trichoderma asperellum*, *Fusarium verticillioides*, plant-fungi interaction, Trichoderma biocontrol activity, maize SWEET transporters

## Abstract

Most Trichoderma species are beneficial fungi that promote plant growth and resistance, while Fusarium genera cause several crop damages. During the plant-fungi interaction there is a competition for sugars in both lifestyles. Here we analyzed the plant growth promotion and biocontrol activity of *T. asperellum* against *F. verticillioides* and the effect of both fungi on the expression of the maize diffusional sugar transporters, the SWEETs. The biocontrol activity was done in two ways, the first was by observing the growth capacity of both fungus in a dual culture. The second one by analyzing the infection symptoms, the chlorophyl content and the transcript levels of defense genes determined by qPCR in plants with different developmental stages primed with *T. asperellum* conidia and challenged with *F. verticillioides*. In a dual culture, *T. asperellum* showed antagonist activity against *F. verticillioides*. In the primed plants a delay in the infection disease was observed, they sustained chlorophyll content even after the infection, and displayed upregulated defense-related genes. Additionally, the *T. asperellum* primed plants had longer stems than the nonprimed plants. SWEETs transcript levels were analyzed by qPCR in plants primed with either fungus. Both fungi affect the transcript levels of several maize sugar transporters differently. *T. asperellum* increases the expression of six SWEETs on leaves and two at the roots and causes a higher exudation of sucrose, glucose, and fructose at the roots. On the contrary, *F. verticillioides* reduces the expression of the SWEETs on the leaves, and more severely when a more aggressive strain is in the plant. Our results suggest that the plant is able to recognize the lifestyle of the fungi and respond accordingly by changing the expression of several genes, including the SWEETs, to establish a new sugar flux.

## 1 Introduction

*Trichoderma* species are free-living fungi that decompose dead organic matter, releasing nutrients that improve soil quality. Also, they are endophytic beneficial fungi that colonize plant roots (Mastouri et al., 2010; Schweiger et al., 2021). During root colonization, *Trichoderma* enhances the plant absorption of nutrients and releases plant growth-promoting molecules improving plant performance and productivity (Contreras-Cornejo et al., 2009; Mastouri et al., 2010; Vinale et al., 2014; López-Coria et al., 2016). Moreover, *Trichoderma* induces metabolic changes in plant tissues (Schweiger et al., 2021). Several species of *Trichoderma* are considered biocontrol agents due to their capacity to antagonize pathogens effectively and to enhance plant defenses against viruses, bacteria, and other fungi (Lorito et al., 2010; Sharma and Sharma, 2020). *Trichoderma* synthesizes a broad spectrum of molecules to fulfill their role as biocontrol, including those that directly attack pathogens, such as bactericides, volatile antibiotics, cell wall degrading enzymes, and proteases (Benítez et al., 2004; Vinale et al., 2008; Druzhinina et al., 2011; Sharma and Sharma, 2020). Contrary to *Trichoderma*, most *Fusarium* species are considered pathogens with detrimental effects on plant development and produce several plant diseases, including *Fusarium* head blight in wheat (Palacios et al., 2021), oat (Ghimire et al., 2020), and barley (Martínez et al., 2021); root rot in soybean (Hafez et al., 2021) and alfalfa (Li et al., 2021); and stem rot and ear rot in maize (Oldenburg et al., 2017). Thus, crop losses caused by *Fusarium* species are an important limitation to food security. In addition, they also impact animal and human health since they release toxins such as fumonisins and aflatoxins (Logrieco et al., 2002; Wild and Gong, 2010).

In both cases, extensive metabolic reprogramming occurs for the host and the microorganism to fight back against each other (Lapin and Van den Ackerveken, 2013; Schweiger et al., 2021). The study of plant–fungi interaction deserves much attention due to their impact on plant productivity. One crucial task that the plant must face when interacting with microorganisms is controlling its sugar partitioning to keep its development and productivity. In the multigene family of transporters, sugar will eventually be exported transporters (SWEETs), which codify for proteins that move a massive flux of sugars in the direction of the concentration gradient. Some of them are located in the plasma membrane, vacuole, and endoplasmic reticulum and are potential targets for microorganisms (Eom et al., 2015; Breia et al., 2021). For instance, a set of *SWEETs* that codify to different sugar specificities located at the plasma membrane are induced in *Arabidopsis* by *Pseudomonas syringae* pv. *tomato* strain DC3000, *Golovinomyces cichoracearum*, and *Botrytis cinerea*, which induce expression of different sets of *SWEETs* (Chen et al., 2010). Enhancement of plasma membrane *SWEET* expression is a strategy for increasing the sugar cell efflux to ensure a constant nutrient supply to the microorganisms, as the latter acts as a sink of carbon nutrients (Chen et al., 2010; Chen et al., 2012). The bacterial pathogen *Xhantomonas oryzae* pv*. oryzae* (Xoo), which causes a severe blight in *Oryza sativa* plants, requires activating specific *Oryza sativa* SWEET (*OsSWEET*) genes to induce the disease’s development (Yang et al., 2006; Antony et al., 2010; Chen et al., 2010; Liu et al., 2011). Plasma membrane sucrose rice transporters, OsSWEET11, OsSWEET13, and OsSWEET14, have promoter regions recognized by the transcription activator-like (TAL) effectors synthesized and injected by *Xanthomonas* into the plant cell (Chu et al., 2006; Römer et al., 2010). Mutants in the TAL effectors cannot induce the expression of SWEETs, causing the disease (Chu et al., 2006; Liu et al., 2011; Yuan et al., 2011).

SWEETs are also a target of beneficial microorganisms. For instance, the beneficial association of *Rhizophagus irregularis* with potato roots produces a significant increase in *SWEET* transcriptional levels. These transporters may be involved in the sugar fluxes that could support mycorrhiza colonization (Manck-Götzenberger and Requena, 2016). In *Medicago truncatula*, the expression of some *SWEETs* is induced in roots colonized by arbuscular mycorrhiza (Kafle et al., 2019).

However, not only SWEETs localized at the plasma membrane are regulated during the plant–microorganism interaction, but in rice *sweet2* mutant plants, they are more susceptible to *Pythium irregulare* infection. OsSWEET2 is a rice glucose transporter located at the vacuoles at the roots; its absence in the mutant plant produces an increase in glucose export, a reduction in the plant growth, and limits the spread of the infection for *P. irregulare* (Chen et al., 2015).

In maize, the SWEET family is composed of 24 members (Sosso et al., 2015; Breia et al., 2021; Liu et al., 2022b; Zhu et al., 2022). Some of them have been characterized and play a key role in plant physiology. For instance, ZmSWEET4c is needed during embryogenesis for starch accumulation in the endosperm (Sosso et al., 2015). The triple mutant *zmsweet13a*, *zmsweet13b*, and *zmsweet13c*, is unable to load the phloem with sugars and reduces overall plant growth (Bezrutczyk et al., 2018). *ZmSWEET15a*, a sucrose transporter, is induced by sucrose and various abiotic stresses. It has been suggested that ZmSWEET15 activity is important for sucrose transport to sink tissues such as the grain, which could be relevant to increasing crop productivity (Liu et al., 2022a). The expression of the ZmSWEET family was recently evaluated during different abiotic stresses such as salt, Cd, and drought (low water potential and ABA) to know the contribution of the *SWEETs* to the plant fitness since sugars are used to deal with the cell stress (Zhu et al., 2022). However, there is no available information in maize about the effect of beneficial or pathogen fungi on *SWEET* expression. Therefore, this work is focused on studying the expression of the most expressed *SWEETs* in the leaves and roots of maize plants (Walley et al., 2016) when interacting with two different fungi lifestyles, *Trichoderma asperellum* and *Fusarium verticillioides*, beneficial and pathogen fungi, respectively. This evidence could contribute to the understanding of sugar partitioning during plant–fungi interaction to improve plant growth and defense responses in this crop.

## 2 Materials and methods

### 2.1 Fungi strains

*T. asperellum* strain HK703 (NRRL50191) was kindly provided by Dr. J. L. Hernández-Mendoza (Centro de Biotecnología Genómica, Tamaulipas, México). *F. verticillioides* MY3 and MY5 strains were kindly provided by Dr. J. Plasencia (Universidad Nacional Autónoma de México). MY3 and MY5 are high and low fumonisin 1 (FB1) producers, respectively (Sánchez-Rangel et al., 2005). Fungi conidia were collected from 2-week-old plate PDA cultures cultivated at 29°C in dark conditions by adding 5 mL of sterile distilled water to the plate cultures incubated for 30 min in orbital agitation. Conidia suspension was collected and centrifuged for 15 min at 13,000 rpm at 4°C. The pellet was washed once in 1.5 mL of sterile distilled water and centrifuged again. Afterward, it was resuspended in 1.0 mL of sterile distilled water. The number of conidia per milliliter was estimated using a hemocytometer. Conidia suspension was stored at 4°C until used (Sánchez-Rangel et al., 2005; López-Coria et al., 2016).

### 2.2 Antagonist fungi activity assay

The antagonistic activities of *T. asperellum* and *F. verticillioides* were tested by the dual culture plate method. *F. verticillioides* and *T. asperellum* were grown on a PDA medium at 25°C for 2 weeks. Three independent replicates were performed for each culture. A section of 1 cm^2^ was taken from each plate and placed on the same fresh PDA plate, 2.5 cm apart from each other. For fungus growth control, PDA plates were inoculated with only one fungus species. Plates were incubated at 28°C for 5 days, and then the growth diameters were measured. Antifungal activities were expressed as the inhibition rate (Dubey et al., 2021): (*rc*–*r*) */ rc* × 100%, where *rc* is the radius of the *F. verticillioides* without the presence of *T. asperellum* and *r* is the radius of the *F. verticillioides* growing with *T. asperellum*.

### 2.3 Seed sterilization

Seeds of *Zea mays* var. Chalqueño were surface sterilized in a 2% (v/v) household bleach (final concentration: 0.12% NaClO) for 2 min and rinsed at least five times with sterile water. For internal sterilization, the seeds were incubated in distilled water at 60°C for 5 min (Glenn et al., 2008; López-Coria et al., 2016). The seed germination percentage was 95% ± 5 at 24 h.

### 2.4 Seed priming treatment and germination

A batch of 200 sterilized seeds was primed for 1.5 h under constant agitation in 200 mL of water containing 1,000 *T. asperellum* conidia/mL (López-Coria et al., 2016) or 6.5 × 10^4^ F*. verticillioides* MY3 or MY5 conidia/mL. No-primed control seeds were incubated for 1.5 h only with water. Seeds were germinated on 1% agar in 20 cm × 20 cm plastic containers at 29°C in dark conditions for 48 h and transplanted either into a hydroponic system or into 10 cm diameter pots filled with sphagnum peat moss (Premier Tech Horticulture, Quebec, Canada).

### 2.5 Hydroponic grown plants

The hydroponic system was carried out using 5-L-square PET bottles cut transversally. The bottom part of each bottle was filled with 2 L of Hoagland solution, prepared by Hoagland and Arnon (1938) (**Supplementary Data**), and constantly aerated through a tube connected to an air pump (Elite 799 Hagen, MA, USA). The upper part of the bottle was placed upside down and attached to the rest of the bottle using plastic film. Five perforations were made in the screw cap to place five seedlings. Seedlings grew for 3 or 30 days in the hydroponic system under greenhouse conditions.

### 2.6 Primed plants

One batch of 30 plants for each treatment: control (C), *T. asperellum* (T) *F. verticillioides* MY3 and *F. verticillioides* MY5 primed (*n* = 30) were transferred from the agar to a 10-cm diameter pot with 150 g of sphagnum peat moss (Premier Tech Horticulture, Quebec, Canada). Pots were watered every other day with tap water for 7 days under greenhouse conditions. The developed leaves were harvested and stored at −80°C until RNA extraction was performed.

### 2.7 Tripartite interaction *Trichoderma*-maize-*Fusarium*

Two batches of 60 control (C) and *Trichoderma* (T) plants were produced as described above, and after 7 days of growing under greenhouse conditions, each treatment was split into two lots. The first lot of C and T plants was infiltrated with water (mock), and the second batch of C and T plants was infiltrated with 6.5 × 10^4^ F*. verticillioides* MY3 conidia/per plant or otherwise indicated in the figures. The infiltration was performed as described by Beernink et al. (2021), using an insulin syringe and loading 10 µL of water or conidia stock solution at 3–4 mm from the coleoptile node. After infiltration, the four lots of treated plants (a) control–mock, (b) primed with *Trichoderma*–mock, (c) control challenged with *Fusarium*, and (d) primed with *Trichoderma* and challenged with *Fusarium*, were grown under greenhouse conditions. Chlorophyll content was measured at 1, 2, 4, 5, and 6 days postinfiltration (dpi). The leaves were then harvested and stored at −80°C until RNA extraction was done.

### 2.8 Sugar content in root exudates

Three batches of five control and five primed hydroponically grown plants, aged 3 and 30 days, were placed in a 1-L Erlenmeyer flask with their roots submerged in 250 mL of deionized water with constant agitation. After 16 h, the solution was filtered through a 0.45-µm membrane and lyophilized. Soluble sugar determination was performed as described by Sánchez-Linares et al. (2012), using 200 mg of the lyophilized powder for ethanol extraction. Glucose (Glu), fructose (Fru), and sucrose (Suc) were determined using an enzymatic assay coupled to NAD^+^ production using the glucose assay reagent (Sigma-Aldrich, Darmstadt, Germany).

### 2.9 Relative chlorophyll content

A nondestructive method was used to measure the chlorophyll concentration in the first leaf of each treated plant. Absorbance was measured in several parts of the leaf using the SPAD-502 Plus Monitor (Konica Minolta Inc., Tokyo, Japan). Values were expressed as SPAD units calculated by the monitor. The determinations were done in two different biological replicas with five plants per replica and at least three measurements per leaf.

### 2.10 RNA extraction and RT-qPCR analysis

RNA was extracted by the guanidine isothiocyanate-phenol-chloroform method using Trizol (Invitrogen, Waltham, MA, USA) according to the manufacturer’s instructions. The quantification of total RNA was carried out using a NANODROP 2000 (Thermo Fisher Scientific Inc., Waltham, MA, USA). The RNA had A260/A280 ratios of 2.0 ± 0.1. The integrity of RNA bands was evaluated in 2% agarose gels by observing the 28S and 18S bands. cDNA synthesis was made using 1 µg of RNA, oligoDT, and the Improm-II™ Reverse Transcription System (Promega, Madison, WI, USA). cDNA was stored at 20°C until use. qRT-PCR was performed in the thermocycler 7500 Real-Time PCR System (Applied Biosystems, Waltham, MA, USA). The reaction mixture contained 10 µL of SYBR Green Master Mix SYBR Green Master Mix (Applied Biosystems, Waltham, MA, USA), 0.15 μL of forward oligonucleotide (20 µM), 0.15 μL of reverse oligonucleotide (20 µM), 2 μL of cDNA, and 7.7 μL of nuclease-free water. For qRT-PCR analysis, the amplification efficiency for each set of primers was calculated after a standard curve was done (Pfaffl, 2001), and all the primers have an efficiency higher than 95%. As control of expression, we used two reference genes: *Zm18S* (Sosso et al., 2015) and *UBQ*. For both genes, the expression levels were unaffected in the different conditions tested in this work. Oligonucleotide sequences for the most expressed *SWEETs* at the leaves and roots (Walley et al., 2016) are listed in **Supplementary Table** **S1**. Relative expression was calculated using the formula (Pfaffl, 2001):


$$\text{Expression ratio}=\;\frac{{\left(E_{target}\right)}^{(\text{Δ}{\text{CP}}_{target}\left(\text{control}-\text{sample}\right)}}{{\left(E_{ref}\right)}^{\text{Δ}{\text{CP}}_{ref}\left(\text{control}-\text{sample}\right)}}\;$$

where *E*_target_ is the efficiency of the gene target, ΔCP_target_ is the Ct value in the control group minus the Ct value of treated group samples, *E*_ref_ is the efficiency of the reference gene, and ΔCP_ref_ is the Ct value of the reference gene in the control group minus the Ct value of the reference gene in the treatment samples. All the determinations were made in two different biological replicates with three technical repetitions.

### 2.11 Statistical analysis

Statistical analysis was performed using the software OriginPro, 2021 Version 9.8.0.200 (OriginLab Corporation, MA. USA). Analysis of chlorophyll content and *SWEET* expression in 3- and 30 day-old roots were made by *t*-test (*p* = 0.05). The other results were analyzed by two-way ANOVA with the Tukey test for significance (*p* = 0.05).

## 3 Results

### 3.1 Direct antagonist activity of *T. asperellum* vs. *F. verticillioides*

**Figure 1:**
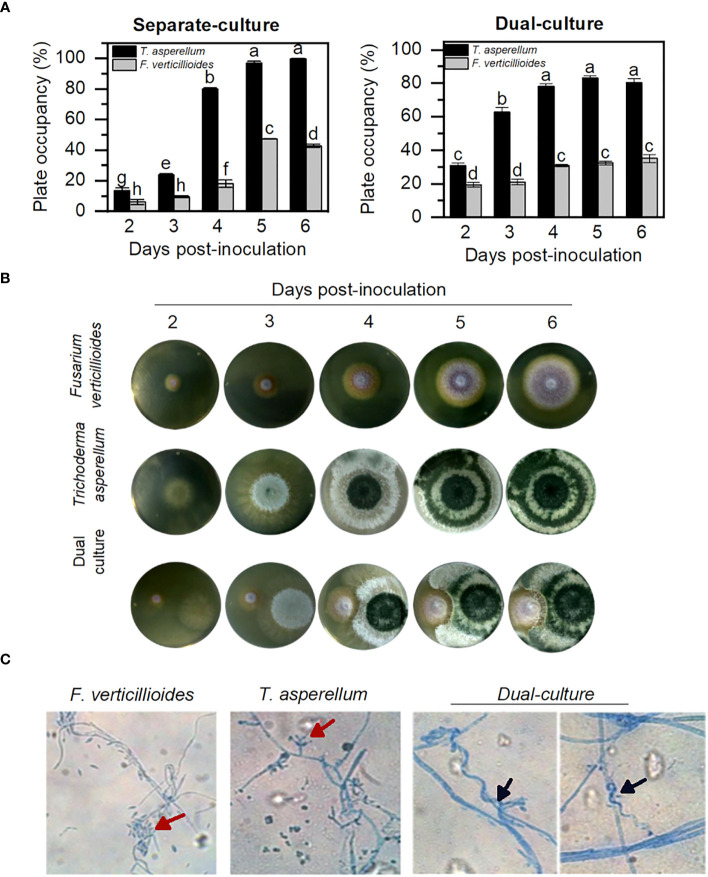
*T. asperellum* delays the growth of *F verticillioides* in a double-culture assay. **(A)** Plate area covered by fungus hyphae in separate plates and plate-area covered by fungus in the co-inoculation plates. Significant differences are pointed out with different letters according to the Tukey test, *p*< 0.05. **(B)** Macroscopic growth of fungus hyphae in PDA plates incubated at 25°C for 6 days. **(C)** Microscopic hyphae view ×40 stained with lactophenol-blue. Red arrows indicate the conidia of each fungus. Black arrows indicate *Trichoderma* haustorium-like formations.

Various members of the *Trichoderma* genus are considered effective biocontrol microorganisms. Here, two essential characteristics were considered to define *T. asperellum* as a biocontrol: its direct antagonist effect over the pathogenic fungus and the indirect activity to reduce the infection *in planta*, which includes the induction of plant systemic resistance (Pocurull et al., 2020; Rivera-Méndez et al., 2020; Saravanakumar and Wang, 2020). The detrimental effect of *T. asperellum* over the pathogen *F. verticillioides* was observed in a dual-culture plate. *T. asperellum* grows faster either alone or in the presence of *F. verticillioides* (**Figures 1A, B**), while, after 5 days of incubation, *F. verticillioides* was unable to cover more than 20% of the plate area in the presence of *T. asperellum*. Microscopic observation of the fungus in the inhibition zone clearly shows a direct interaction between the two fungus species, with *T. asperellum* hyphae coiling around *F. verticillioides* hyphae (**Figure 1C**), the so-called mycoparasitism. Both antagonistic activities interfere with the pathogen’s survival and can be used when the fungi are in the same niche, such as the rhizosphere.

### 3.2 *T. asperellum* enhances maize resistance to *F. verticillioides* infection

**Figure 2:**
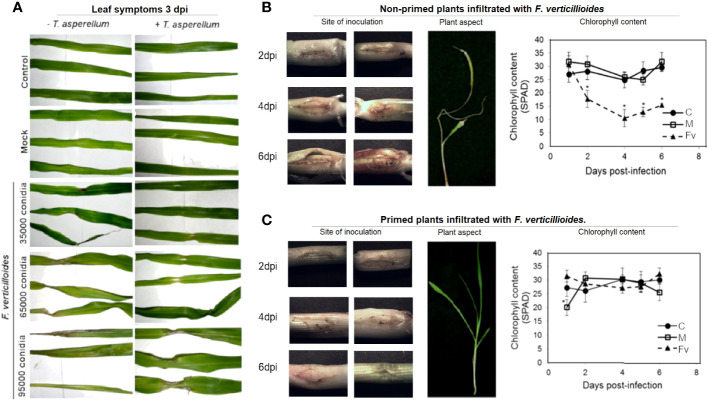
Symptoms of *F verticillioides* infection appear 3 days after infiltration in nonprimed *T. asperellum* maize plants. **(A)** Leaf appearance after 3 days of *F verticillioides* infiltration. Noninfiltrated plants of nonprimed or primed with *T. asperellum* (*+T. asperellum*) plants are the control plants; mock plants were infiltrated with water, and *F verticillioides* infiltrated plants with different amounts of *F verticillioides* MY3 conidia, as indicated. **(B)** Representative photographs of plant stems from nonprimed plants showing the *F verticillioides* infiltration site on different days postinfiltration. The experiment was repeated five times with two replicas each. Moreover, the plant aspect after 3 days of 6.5 × 10^4^ conidia *F verticillioides* infiltration and chlorophyll content of nonprimed plants along 6 days postinfection are shown. **(C)** Plant stems from primed plants show the *F verticillioides* infiltration site on different days postinfiltration. Plant aspect after 3 days of 6.5 × 10^4^ conidia *F verticillioides* infiltration and chlorophyll content of nonprimed plants along 6 days postinfection. C, control; M, mock; Fv, infiltration with 6.5 × 10^4^ F*. verticillioides* conidia. Symbols represent the average of two independent biological samples with *n* = 12 ± SD. Asterisks indicate significant differences according to *t*-test, *p* = 0.05.

To ensure that the seeds used in the following experiments were adequately disinfected, we germinated disinfected seeds on PDA agar plates (**Supplementary** **Figure S1**). No fungus or other contamination was observed. To demonstrate the indirect biocontrol activity of *T. asperellum*, *Trichoderma*-primed maize seeds were germinated and grown for 9 days and then infiltrated at the stem with different amounts of conidia of high pathogenic *F. verticillioides MY3* strain or water as a mock to determine the biocontrol activity of *Trichoderma* against *F. verticillioides in planta*. Nonprimed plants (**Figure 2A** **;** *−T. asperellum*) developed infection symptoms after 3 days postinfection with 3.5 × 10^4^, 6.5 × 10^4^, and 9.5 × 10^4^ conidia of *F. verticillioides* MY3. However, the primed plants developed minor symptoms at the higher conidia content (**Figure 2A**; + *T. asperellum*). Infiltration of 6.5 × 10^4^ F*. verticillioides* conidia at the stems of nonprimed plants maintains an open wound with pink color at the zone of infiltration during 6 dpi, whereas the primed plants have a close wound with no symptoms of infection (**Figures 2B, C**). The leaves developed a pale green or yellow coloration (**Figure 2B**; +*T. asperellum* and **Figure 2C**), but not in *T. asperellum* primed plants and then infected with *F. verticillioides* (**Figure 2C**; +*T. asperellum*; **Figure 2C**). Priming treatment prevents the decrease of chlorophyll after 2 days of infection with 6.5 × 10^4^ F*. verticillioides* conidia (**Figure 2C**), a healthy plant symptom.

**Figure 3:**
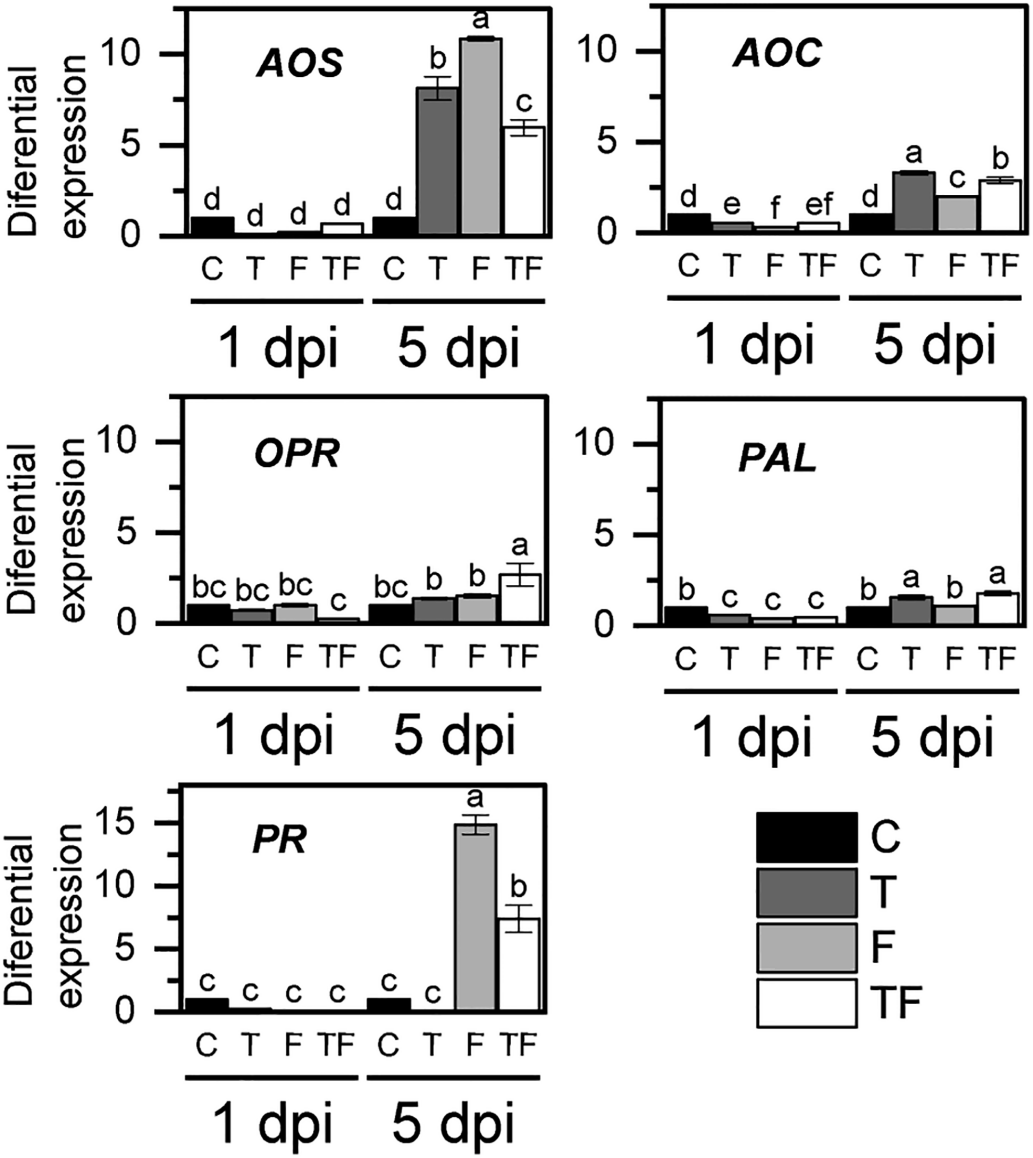
Relative expression of plant immune defense genes. C, control; T, primed with *T. asperellum*; F, infiltrated with *F. verticillioides*; TF, primed with *T. asperellum* and infiltrated with *F. verticillioides*. Bars represent the average relative expression in triplicate from two independent biological samples (*n* = 6), normalized with *Zm18S* and nonprimed plant expression ± SD. Different letters correspond to differences of significance at *p* = 0.5, according to the Tukey test. *AOS*, allene oxide synthase; *AOC*, allene oxide cyclase; *OPR*, 12-oxo-phytodienoic acid reductase; *PAL*, phenylalanine ammonium lyase; PR, pathogenesis-related proteins.

To determine if *T. asperellum* affected the *F. verticillioides* maize defense responses, we evaluated the expression of defense response genes at 1 dpi and 5 dpi (**Figure 3**), times at which both fungi have been shown to elicit responses in the plant (Bartholomew et al., 2019; Anisimova et al., 2021; Pacheco-Trejo et al., 2022). We evaluated the induced systemic response (ISR) by detecting the effect on the expression of jasmonate (JA) biosynthesis pathway key enzyme-coding genes (Van der Ent et al., 2009): allene oxide synthase (*AOS*), allene oxide cyclase (*AOC*), and 12-oxo-phytodienoic acid reductase (*OPR*). High expression levels were observed at 5 days of *F. verticillioides* postinfection (**Figure 3**), particularly *F. verticillioides* infection and *T. asperellum* priming enhanced *AOS* expression. However, *AOS* expression levels decreased by half in the *T. asperellum*-primed plants infected with *F. verticillioides* plants (TF) compared with nonprimed plants infected with *F. verticillioides* (F).

Plant interaction with microorganisms induced not only the ISR pathway but also systemic acquired resistance (SAR), which is associated with salicylic acid (SA) accumulation as a consequence of the activation of the phenylalanine ammonium lyase (*PAL*) gene transcription (Mitra et al., 2020). Here, we observed a significant increase in the transcription of *PAL* in T and TF treatments. In addition, SA also induces the expression of several defense genes, such as pathogenesis-related proteins (PR). The expression of *PR* in nonprimed plants infected with *F. verticillioides* (F) showed a 15-fold increase when compared to control plants (C), but in TF treatment, *PR* expression was only seven times higher than in control plants (**Figure 3**).

### 3.3 *Trichoderma asperellum* promotes maize plant growth and increases *SWEETs* expression in aerial tissues and roots

**Figure 4:**
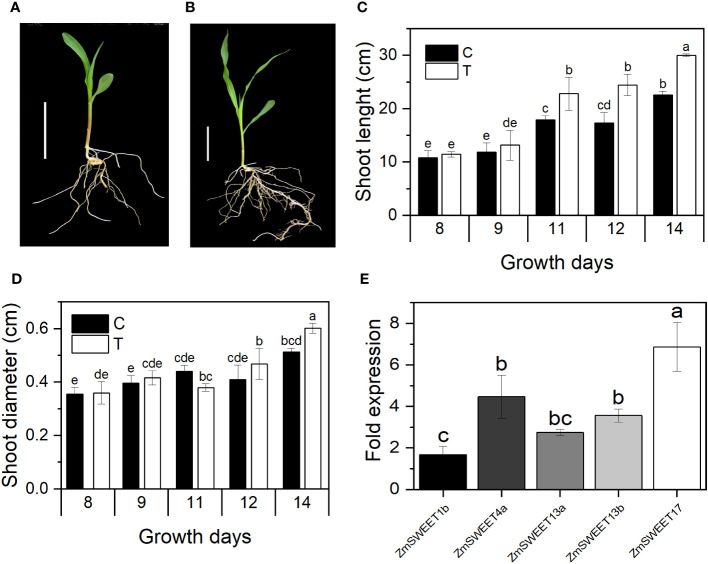
Plant growth improvement by *T. asperellum* seed priming. **(A)** A representative image of nonprimed plant. **(B)** A representative plant primed with *T. asperellum*. *n* = 30; bar = 5 cm. **(C)** Shoot length. **(D)** Shoot diameter of control **(C)** and primed plants (T). **(E)** RT-qPCR analysis of *ZmSWEET* relative expression in 14-day-old aerial tissues. Bars represent normalized relative expression. Error bars indicated the ± SD of the mean of three replicates from two independent biological samples, *n* = 6. Different letters indicate significant differences according to the Tukey test, *p* = 0.05.

Seed priming treatment with 1,000 *T. asperellum* conidia/mL (T) promoted plant development. After 11 days of growth under greenhouse conditions, stem enlargement and diameter increased in primed plants (**Figures 4C, D**). There was no change in the primary root length. However, they showed more development of secondary roots in primed plants than in control plants (**Figures 4A, B**).

According to the MaizeGDB RNAseq database (Walley et al., 2016), in the B73-maize aerial tissue, *ZmSWEET1b*, *ZmSWEET4a*, *ZmSWEET13a*, *ZmSWEET13b*, and *ZmSWEET17* are the main expressed isoforms. Their expression was determined here by RT-qPCR in 14-day-old plants. Relative expression was double normalized to constitutive gen *Zm18S* expression and *SWEET* expression in nonprimed plants. The mRNA levels of the five SWEET isoforms were up at least 1.6 times due to *T. asperellum* priming treatment. The most significant increment was found in the ZmSWEET17 mRNA, which increased 6.8 times in primed plants (**Figure 4E**).

**Figure 5:**
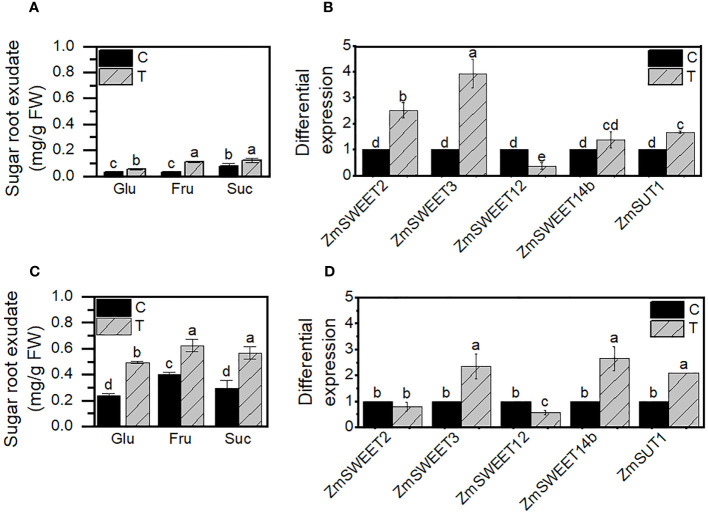
Sugars exudated by primed roots and *ZmSWEET* root expression. **(A)** Sugar root exudates and **(B)** differential *SWEET* expression in roots at 3 days of growth. **(C)** Sugar root exudates and **(D)** differential *SWEET* expression in roots at 30 days of growth. Control **(C)** or primer plants with *T. asperellum* (T). Error bars indicate the ± SD of the mean of three replicates of two independent biological samples (*n* = 6). Different letters indicate significant differences according to the Tukey test, *p*< 0.05.

Variation in sugar transporter transcript levels at the leaves due to *Trichoderma* priming could indicate a modification in the plant sugar allocation to improve plant growth and nourish the fungi in the rhizosphere since *T. asperellum* is a plant root colonizer. To evaluate if that was the case, we determined the amount of soluble sugars Glu, Fru, and Suc in the root exudates of nonprimed (C) and primed (T) plants at 3 and 30 days old. Two different developmental stages were chosen to evaluate the *SWEET* expression, embryonic roots from 3 days and mature radicular system from 30-day-old plants. Suc and Fru were the most abundant sugars in the root exudates. Glu, Fru, and Suc increased more than 200 times with the primed treatment (T), with higher levels in roots of 30-day-old than in 3-day-old roots (**Figures 5A–C**).

Also, we evaluated if the mainly expressed isoforms in maize roots, according to the MaizeGDB RNAseq database (Walley et al., 2016), were expressed differently in primed plants’ roots. The priming treatment enhances the expression of two of the four *SWEETs* analyzed, *ZmSWEET2* and *ZmSWEET3*, in roots within 3 days of plant growth (**Figure 5B**), and *ZmSWEET3* and *ZmSWEET14b* at 30-day-old roots compared to their control group (**Figure 5D**). The effect of *Trichoderma* in the *SWEET* expression lasts at least 30 days.

Along with the *SWEET* transcription increment, the expression of SUT1, the main sucrose transporter in maize (Slewinski et al., 2010), which drives the sucrose accumulation into the cell, was also induced by the priming treatment in both young and old roots.

### 3.4 *F. verticillioides* MY3 reduces the expression of several *SWEETs* in the leaves of nonprimed plants

Among *F. verticillioides* species, some strains can synthesize high levels of mycotoxins, such as fumonisin 1 (FB1). *F. verticillioides* capacity of FB1 production is associated with its aggressiveness to infect plant tissues (Galeana-Sánchez et al., 2017). Here, we explored the effect of two *F. verticillioides* strains: MY3, a high FB1 producer, and MY5, a low FB1 producer (Sánchez-Rangel et al., 2005) on the *SWEET* expression in leaves. For plant infection, we used the conidia concentration of both strains that kept the plants alive (**Supplementary Figure S2**).

**Figure 6:**
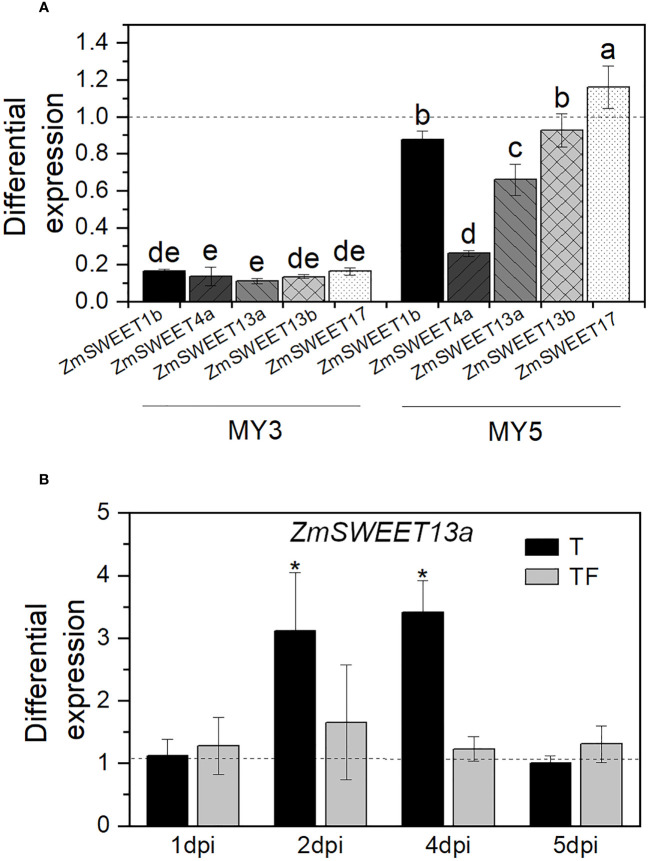
Expression of *ZmSWEET* in plants infected with *F verticillioides*. **(A)** *ZmSWEET* expression in plants infected with *F. verticillioides* MY3 or MY5 strains. Bars represent relative expression normalized with Zm18S, and noninfected plant expression is indicated with the dashed line ± SD of the mean of three replicates of two independent biological samples, n = 6. Different letters indicate significant differences according to the Tukey test, p=0.05. **(B)** Relative expression of *ZmSWEET13a* in *T. asperellum*-primed plants (T) or in primed plants plus *F. verticillioides* MY3 infection (TF), on different days postinfection (dpi). Bars represent relative expression normalized with Zm18S and nonprimed plants as a control (dashed line). Error bars indicated the ± SD of the mean of three replicates of two independent biological samples, n = 6. Asterisks indicate significant differences according to the Tukey test, p = 0.05.

We observed that *F. verticillioides* MY3, which is the most aggressive strain, reduces an average of 87% of the expression of the *ZmSWEET*s (**Figure 6A**). In contrast, the less pathogenic strain MY5 induced a variable response where the mRNA level of *ZmSWEET4a* was the most decreased, followed by *ZmSWEET*13a, *ZmSWEET*1b, and *ZmSWEET*13b.

*ZmSWEET13a*, *ZmSWEET13b*, and *ZmSWEET13c* are members of the ZmSWEET13 family and are one of the most critical transporters in maize leaves; they are mainly involved in phloem loading (Bezrutczyk et al., 2018). Therefore, we analyzed the expression pattern of one of the members, *ZmSWEET13a*, in the leaves of primed plants. Compared to the expression in nonprimed plants (dashed line in **Figure 6B**), priming treatment (T) significantly enhances the expression of *ZmSWEET13a*. However, the expression in primed plants was then infected with *F. verticillioides* (TF) does not significantly change compared to nonprimed plants after infection. Thus, the above demonstrates that the priming treatment with *T. asperellum* did not downregulate the *ZmSWEET13a* expression when the pathogen *F. verticillioides* infects the primed plant; on the contrary, it transiently enhances its expression.

## 4 Discussion

The most common strategies used to control plant fungal diseases are disease-resistant crop cultivation and crop rotation (Savary et al., 2012). However, herbicides like glyphosate also exhibit plant fungal disease control in wheat and soybean glyphosate-resistant plants (Feng et al., 2005; Claus et al., 2023). Extensive evidence, however, demonstrated that pathogen biocontrol using beneficial microorganisms such as *Trichoderma* species could enhance plant health since the beneficial fungus competes against pathogens in the rhizosphere (Stummer et al., 2022), induces plant immune responses (Sood et al., 2020), and is available to modify the metabolomic plant context (Schweiger et al., 2021). Here, we demonstrated that *T. asperellum* could promote young maize plants’ growth and act as a biocontrol agent against *F. verticillioides*, and modify the transcription of several *SWEET* transporters in roots and leaves and the sugar content at the roots.

Priming seeds with beneficial microorganisms is a pregerminative treatment to enhance plant growth. Some widely used microorganism genera for priming seeds are *Aspergillus*, *Azospirillum*, *Bacillus*, *Rizophagus*, *Glomus*, and *Trichoderma*, among others (Arora et al., 2020). For example, tomato seeds treated with *T. harzianum* T-22 germinated earlier, and their radicle grew longer even when the fungus did not colonize the seed embryo, meaning that the fungus exudates have growth-promoting activity (Mastouri et al., 2010). Germinated maize seeds primed with *T. asperellum* show longer mesocotyls and larger radicles with higher root hairs (López-Coria et al., 2016). This increase correlates with the increased activity of a key enzyme that promotes cell elongation, the plasma membrane H^+^-ATPase (López-Coria et al., 2016).

Several secondary metabolites exudating from *Trichoderma* have been proposed to induce plant growth, such as auxins, harzianic acid, koniginin A, 6-pentyl-α-pyrone (Lorito et al., 2010; Vinale et al., 2014). In addition, *T. virens* synthesizes indole-3-acetic acid, indole-3-acetaldehyde, and indole-3-ethanol, auxin-related compounds, promoting lateral root and shoot development (Contreras-Cornejo et al., 2009). *Trichoderma* also affects the transcription of some plant genes (Sood et al., 2020; Schweiger et al., 2021). All the changes in the plant promoted by *Trichoderma* species could drive a modification in carbon plant allocation, which can be beneficial for both the microorganism and the plant. Here, we observed that priming treatment increases the expression of *ZmSWEET17*, *ZmSWEET4a*, *ZmSWEET13a*, and *ZmSWEET13b* in the leaves of 14-day-old plants. From them, only the ZmSWEET13 subfamily has been characterized as a plasma membrane sucrose transporter necessary for phloem loading in maize plants (Bezrutczyk et al., 2018). ZmSWEET4a remains as one putative plasma membrane glucose transporter and ZmSWEET17 as one putative vacuolar fructose transporter (Eom et al., 2015). The increase in the transcription levels of *SWEET* transporters in leaves could lead to a rise in the sugar transport activity that could not only be beneficial for plant growth but could also increase the carbon flux from leaves to roots. The exudated sugars could sustain *Trichoderma* establishment at the rhizosphere.

In roots, *ZmSWEET2*, *ZmSWEET3*, *ZmSWEET12a*, and *ZmSWEET14b* are the most expressed according to RNAseq analysis (Walley et al., 2016). Our results indicate that SWEETs’ expression levels change throughout the development of roots and show differential responses to priming treatment. The increase in the expression of *ZmSWEET2* and *ZmSWEET3* in the primed plants does not seem to lead to a significant increase in the sugar content exudated by the 3-day-old roots, which could be explained by the increase in the SUT1 transcription level since SUT1 is a transporter that supports the intracellular accumulation of sucrose (Slewinski et al., 2010), but it could also mean that the microorganism is using the sugar located at the rhizosphere. In addition, as far as we know, there is no information about the intracellular location of ZmSWEET2 and ZmSWEET3. In rice, OsSWEET2 is located at the vacuole (Chen et al., 2015); if that is the case for ZmSWEET2, the increase in its expression could reduce the sugar at the cytoplasm due to the increase of the sugar flux into the vacuole, limiting the available sugars to be exported at the apoplast and used by the microorganisms. In 30-day-old roots, there was a significant increase in soluble sugars exudated by the roots that could be due to ZmSWEET3 and ZmSWEET14b efflux activity. ZmSWEET14b could be a plasma membrane sucrose transporter since it is closely related to the ZmSWEET13 subfamily (Liu et al., 2022b; Zhu et al., 2022); if that is the case, an enhanced transcription could lead to an increase in the protein at the plasma membrane and the rise of the sugar efflux to nourish the fungi at the 30-day-old roots. Nevertheless, since we only evaluated the expression of the *SWEET* isoforms reported in the root RNAseq study of Walley et al. (2016), it is possible that other *SWEET* not considered here could be involved in the root sugar secretion, such as ZmSWEET1a, ZmSWEET4a, and ZmSWEET13c, that were found to have high expression in primary roots (Zhu et al., 2022). Also, the amount of SWEET expressed at the membranes remains to be determined. Our results indicate that during the interaction between maize and *T. asperellum*, the increment of sugars exudated by mature roots can support the fungi nourishment, where *ZmSWEET3* and *ZmSWEET14b* could be involved. As discussed, the expression of *SWEETs* is not clear in plant–microorganism interactions since, in different scenarios, it was reported an upregulation or downregulation of *SWEETs*, indicating to be a specie-specific response, and we can also suggest that it is developmentally regulated.

Members of the SWEET family in maize are also susceptible to being altered by abiotic stress. Zhu et al. (2022) analyzed the SWEET family in maize and their expression pattern by abiotic stress, or ABA. Several SWEETs were upregulated by ABA, such as *ZmSWEET1a*, *ZmSWEET4c*, *ZmSWEET14b*, *ZmSWEET15b*, *ZmSWEET16*, and *ZmSWEET17a*, and the other four *SWEETs* were induced by abiotic stress but not by ABA. Abiotic stress such as drought or high salt concentration alters the intracellular and tissue sugar allocation to deal with the deleterious effect of the stress. These results suggest that the content is vital to the plant in stressful situations (Jeandet et al., 2022).

Several mechanisms have been suggested regarding the biocontrol activity of *Trichoderma* species against pathogenic fungi. Recognition of the pathogen by the beneficial fungus leads to the exudation of metabolites and enzymes with antibiosis and lytic activities. Such molecules enhance mycoparasitism and the competition for space and nutrients (Köhl et al., 2019; Sharma and Sharma, 2020; Schweiger et al., 2021), which *T. asperellum* may display when interacting with *F. verticillioides* since we detected *T. asperellum* hyphae coiling around the *F. verticillioides* hyphae, and also the reduction of *F. verticillioides* growth when both fungi were loaded in the same plate. He et al. (2019) found that *T. asperellum*-treated soil reduced maize stalk and ear rot produced by *Fusarium*, and the content of FB1 and deoxynivalenol in the ear and grain was reduced at basal levels. *T. asperellum* was also able to reduce and select a variety of endophytic microorganisms in a mature plant. The authors suggest that the contribution of *T. asperellum* to plant fitness was not only to induce the defense response against the pathogen but also to select a specific set of endophytic microorganisms that can also compete with *Fusarium*. In this work, the study was made with sterile seeds, in which *T. asperellum* enhances the defense response and reduces *F. verticillioides* symptoms in the stem and leaves; further work is needed to determine which molecules or microorganisms could synergistically help *Trichoderma* induce the plant response to a specific pathogen attack.

Additionally, *Trichoderma* triggers the plant immune system response (Druzhinina et al., 2011). An unknown mechanism disrupts the early response, which leads to the success of the mutualistic plant–*Trichoderma* interaction. However, a later defense response could be produced when a second infection by a different microorganism, wound (herbivores), or salt stress is perceived (Ankala et al., 2013). Here, we observed that *T. asperellum* not only triggers the induced immune response (or ISR) by the JA/ethylene pathway but also triggers the SAR response only when *F. verticillioides* infects the plant. SAR induction has been reported for *Trichoderma* species when associated with pathogens (Druzhinina et al., 2011). However, it is a response that could be different between plant and fungus species. Chen et al. (2021) found that some isoforms of *PAL* are not enhanced in dual *Trichoderma harzianum* and *Fusarium oxysporum* interaction with *Radix pseudostellariae*, contrary to *PR* gene expression. Ben Amira et al. (2017) observed that *PAL*, *PR*, and *AOC* transcripts increase with the interaction of both *T. harzianum* and *F. solani* in olive trees. *T. asperellum* improves maize performance against *Fusarium verticillioides* and induces maize *SWEET* sugar transport expression adjustment, resulting in a high sugar root exudation. However, these adjustments do not compromise plant growth, as shown by the chlorophyll content and plant performance.

In relation to the effect of the hemibiotrophic pathogen *F. verticillioides* on plant performance, we used MY3 and MY5 strains with conidia concentration that keep the plants alive and growing and with a sustained chlorophyll content, features that suggest that both strains are in the biotrophic cycle. However, even though the plants were asymptomatic, they experimented with different reduction profiles of the expression of *ZmSWEET1b*, *ZmSWEET4a*, *ZmSWEET13a*, *ZmSWEET13b*, and *ZmSWEET17* in aerial tissue: higher reduction with the high FB1 producer strain, MY3, and less abrupt reduction with the low FB1 producer strain, MY5. Chong et al. (2014) reported that two biotrophic pathogens, *Erysiphe necator* and *Plasmopara viticola*, do not induce the *SWEET* expression in *Vitis vinifera*, contrary to the necrotrophic fungus *Botrytis cinerea*. These results agree with our observations. It is accepted that a biotrophic pathogen’s nutrition strictly depends on the supply of organic carbon and nitrogen metabolites from living host tissue (Divon and Fluhr, 2007), and a necrotrophic organism lives on dead tissues. However, maize plants can detect and change the *SWEET* expression slightly differently if it is interacting with a high-pathogenic strain or not, even when both are in their biotrophic cycle, suggesting that fungi are expressing different molecules that could be related to their pathogenic potential and the plant can sense and switch on a different set of responses. Therefore, the decrease in *ZmSWEET* transcripts may reduce the internal plant sugar flux, preparing the defense against the pathogen.

Additionally, the presence of both beneficial and pathogenic fungi at the same time results in high but transitory expression of *ZmSWEET13a*, a member of the ZmSWEET13 subfamily involved in the apoplastic phloem loading in maize (Bezrutczyk et al., 2018). An increase in *ZmSWEET13a* expression could be related to the sugar reallocation necessary to support plant–*Trichoderma* interaction. *MtSWEET1b* is highly expressed in the peri-arbuscular membrane of roots colonized by the fungus *Rhizophagus irregularis*, and its overexpression promotes the growth of intraradical mycelium. However, mycorrhization is not affected if the SWEET transporter loses its function (An et al., 2019).

It would be simplistic to point out that microorganisms act only as new sinks for the plant because the plant needs to recognize the fungus interacting with it to set up its biochemical and genetic reprogramming before giving access to its nutrients. Here, we showed that the maize plant is able to modify the *SWEET* expression differently depending on the lifestyle of the fungi. Still, it could also affect other types of sugar transporters, which are essential to regulating the plant carbon partitioning to continue its plant development.

## Data availability statement

The raw data supporting the conclusions of this article will be made available by the authors, without undue reservation.

## Author contributions

ML-C: Conceptualization, Data curation, Formal Analysis, Investigation, Methodology, Writing – original draft. FG-C: Investigation, Formal Analysis, Writing – review & editing. RC-G: Formal Analysis, Investigation, Methodology, Writing – review & editing. DM-C: Formal Analysis, Investigation, Methodology, Writing – review & editing. TS-S: Investigation, Methodology, Writing – review & editing. JA-R: Formal Analysis, Investigation, Methodology, Writing – review & editing. BK-D: Formal Analysis, Methodology, Project administration, Writing – review & editing. SS-N: Conceptualization, Formal Analysis, Funding acquisition, Supervision, Writing – review & editing.
