# Supplementary material for: Maize plant expresses SWEET transporters differently when interacting with Trichoderma asperellum and Fusarium verticillioides, two fungi with different lifestyles
Source: Front Plant Sci. 2023 Sep 27;14:1253741. doi: 10.3389/fpls.2023.1253741 (PMC10565004; doi:10.3389/fpls.2023.1253741)
Supplement: Supplementary file 1 [file DataSheet_1.docx]

Supplementary Material

Maize plant modulates SWEET transporters self-expression when independently facing *Trichoderma asperellum and Fusarium verticillioides*, two fungi with different lifestyles.

**Montserrat López-Coria^1^, Fernando Guzmán-Chávez^1^, Roberto Carvente-García^1^, Daniela Muñoz-Chapul^1^, Tomás Sánchez-Sánchez^1^, Juan Manuel Arciniega-Ruíz^1^, Beatriz King-Díaz^1^, Sobeida Sánchez-Nieto^1^***†

^1^Dpto. de Bioquímica, Facultad de Química, Conjunto E. Universidad Nacional Autónoma de México, Cd. Universitaria, Coyoacán. México 04510, D.F., México. +52(55)56225952.

*** Correspondence:** Sobeida Sánchez Nieto. sobeida@unam.mx

# Supplementary Data

**1.1. Hoagland solution composition.**

| Reagent | Formula | Final concentration |
| --- | --- | --- |
| Monopotassium phosphate | KH_2_PO_4_ | 1 x 10^-3^ M |
| Potassium nitrate | KNO_3_ | 5 x 10^-3^ M |
| Calcium nitrate tetrahydrate | Ca(NO_3_)_2_•4H_2_O | 5 x 10^-3^ M |
| Magnesium sulfate heptahydrate | MgSO_4_•7H_2_O | 5 x 10^-3^ M |
| Micronutrient solution | | |
| Boric acid | H_3_BO_3_ | 4.6 x 10^-5^ M |
| Manganese chlorid tetrahydrate | MnCl_2_•4H_2_O | 9.1 x 10^-6^ M |
| Zinc sulfate heptahydrate | ZnSO_4_•7H_2_O | 9.7 x 10^-7^ M |
| Copper sulfate pentahydrate | CuSO_4_•5H_2_O | 3.2 x 10^-7^ M |
| Sodium molibdate dihydrate | Na2MoO4•2H_2_O | 1.2 x 10^-7^ M |
| Iron chelate | Fe-EDTA | 0.015 g/mL |

# Supplementary Table and Figures

## Supplementary Tables

**Supplementary Table 1. List of primers used for the expression analysis by RT-PCR and RT-qPCR.**

| Name | Sequence | Amplicon length (bp) | Amplification efficiency (Pfaffl, 2001) |
| --- | --- | --- | --- |
| *ZmSWEET1b*  NM_001154214.1 | Fw 5’-TCCATATAAGCGCAAGCAGACA-3’ | 151 | 2.1838 |
|  | Rv 5’-CAGAACGTAGGCACTGGGG-3’ |  |  |
| *ZmSWEET2*  GRMZM2G324903 | Fw 5’-AGGCTCAAGGTCTCTGCTCT-3’ | 163 | 1.9459 |
|  | Rv 5’-TGACGATTGACATGGGGGAC-3’ |  |  |
| *ZmSWEET3*  GRMZM2G060974 | Rv 5’-CTTCCAGATGCGGATGAACG-3’ | 158 | 2.0559 |
|  | Rv 5’-AACAGTTGGGTCGCTGCTAT-3’ |  |  |
| *ZmSWEET4a*  NM_001175008.1 | Rv 5’-TGACGATTGACATGGGGGAC-3’ | 177 | 2.1232 |
|  | Rv 5’-CTTCCAGATGCGGATGAACG-3’ |  |  |
| *ZmSWEET12a*  GRMZM2G133322 | Fw 5’-GACCTGACTATGATTGCTGC-3’ | 114 | 2.0134 |
|  | Rv 5’-GCGTCTACTTGTACCGTGGT-3’ |  |  |
| *ZmSWEET13a*  NM_001155615.1 | Fw 5’-CGTGGAGTACATGCCCTTCT-3’ | 151 | 1.9371 |
|  | Rv 5’-CACGTAGAGCACCCATCTGGT-3’ |  |  |
| *ZmSWEET13b* NM_001148182.1 | Fw 5’-ACAAATACGTCGCGCTACCA-3’ | 361 | 2.0957 |
|  | Rv 5’-GCTTGCTTGCGATGATGGAG-3’ |  |  |
| *ZmSWEET14b*  GRMZM2G015976 | Fw 5’-GTCATCGAGACCCTCTAC-3’ | 200 | 1.9807 |
|  | Rv 5’-ACGAAGACGCTAACGGAGAA-3’ |  |  |
| *ZmSWEET17* DAA54392.1 | Fw 5’-TCATGCCGTTCTTCCTATCC-3’ | 195 | 2.0139 |
|  | Rv 5’-CAAAGAAATAGCGTCGCCTC-3’ |  |  |
| *PR*  NM_001159109.1 | Fw 5’-GGCCACTACACCCAGATCAT-3’ | 200 | 1.9692 |
|  | Rv 5’-TGGGACAGCAAGAGACACAG-3’ |  |  |
| *PAL*  [NM_001254868.1](http://www.ncbi.nlm.nih.gov/entrez/viewer.fcgi?db=nucleotide&id=363543166) | Fw 5’-CATGTCGTCCACCTACATCG-3’ | 171 | 1.9932 |
|  | Rv 5’-ATCAGCTCCTTCTCGCTGAA-3’ |  |  |
| *AOS*  NM_001111774.1 | Fw 5’-GTGGACGTGACAGAGTGTCC-3’ | 152 | 1.9553 |
|  | Rv 5’-CAACATGCCATCCTGCATTA-3’ |  |  |
| *AOC*  Ankala et al., 2013 | Fw 5’-GCTACGAGGCCATCTACAGC-3’ | 160 | 1.9475 |
|  | Rv 5’-GGGAAGACGATCTGGTTGAG-3’ |  |  |
| *OPR*  Ankala et al., 2013 | Fw 5’-TATATCAGCCGGGTGGTTCT-3’ | 184 | 1.9553 |
|  | Rv 5’-CCTGCTTTGATGGCGTTTAT-3’ |  |  |
| *UBQ*  (Reference gene)  Zhang et al., 2012 | Fw 5’-ATCTTTGTGAAGACCCTCAC-3’ | 216 | 2.0465 |
|  | Rv 5’CCTAAGGCGCAGCACCAAGT-3’ |  |  |
| *18S*  (Reference gene) | Fw 5’-CCATCCCTCCGTAGTTAGCTTCT | 152 | 1.9970 |
|  | Rv 5’-CCTGTCGGCCAAGGCTATATAC |  |  |
| *ZmSUT1*  GRMZM2G034302 | Fw 5’-TCCTCTGGCTCCACAAACAAC-3’ | 463 | 2.0105 |
|  | Rv 5’- ACGAGCTGAATCCTAGAACGA-3’ |  |  |

## Supplementary Figures


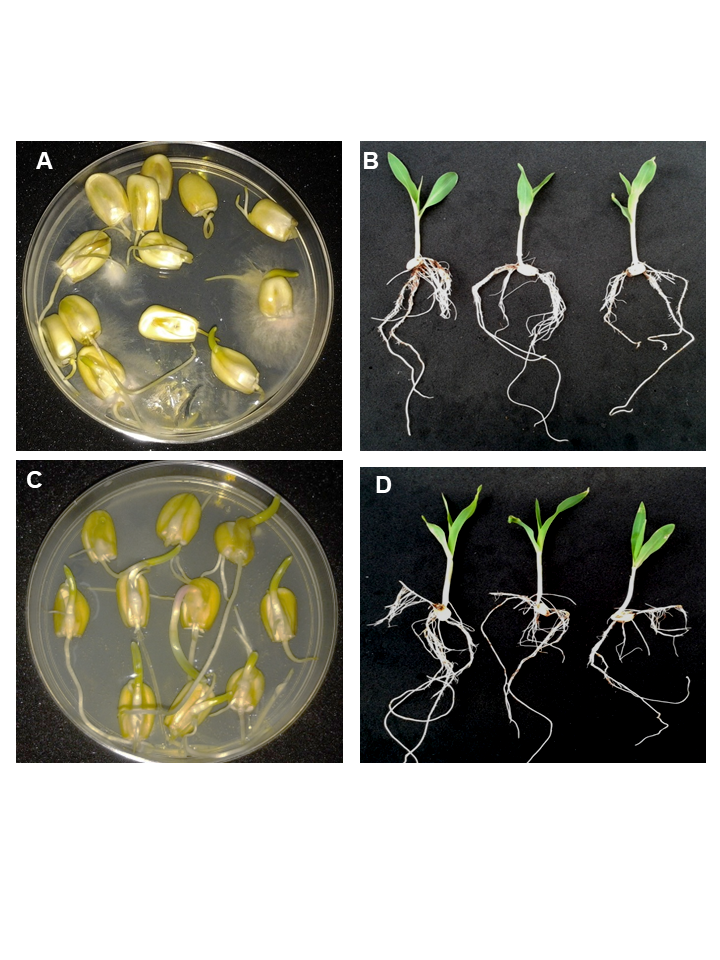


**Supplementary Figure 1. Effect of disinfection treatment in seed germination and development. (A)** Germinated seeds after surface disinfection treatment with 0.12% NaOCl. **(B)** Seedlings from surface seed disinfected. **(C)** Germinated seeds after surface and internal disinfection. **(D)** Seedlings from seeds with both disinfection treatments.


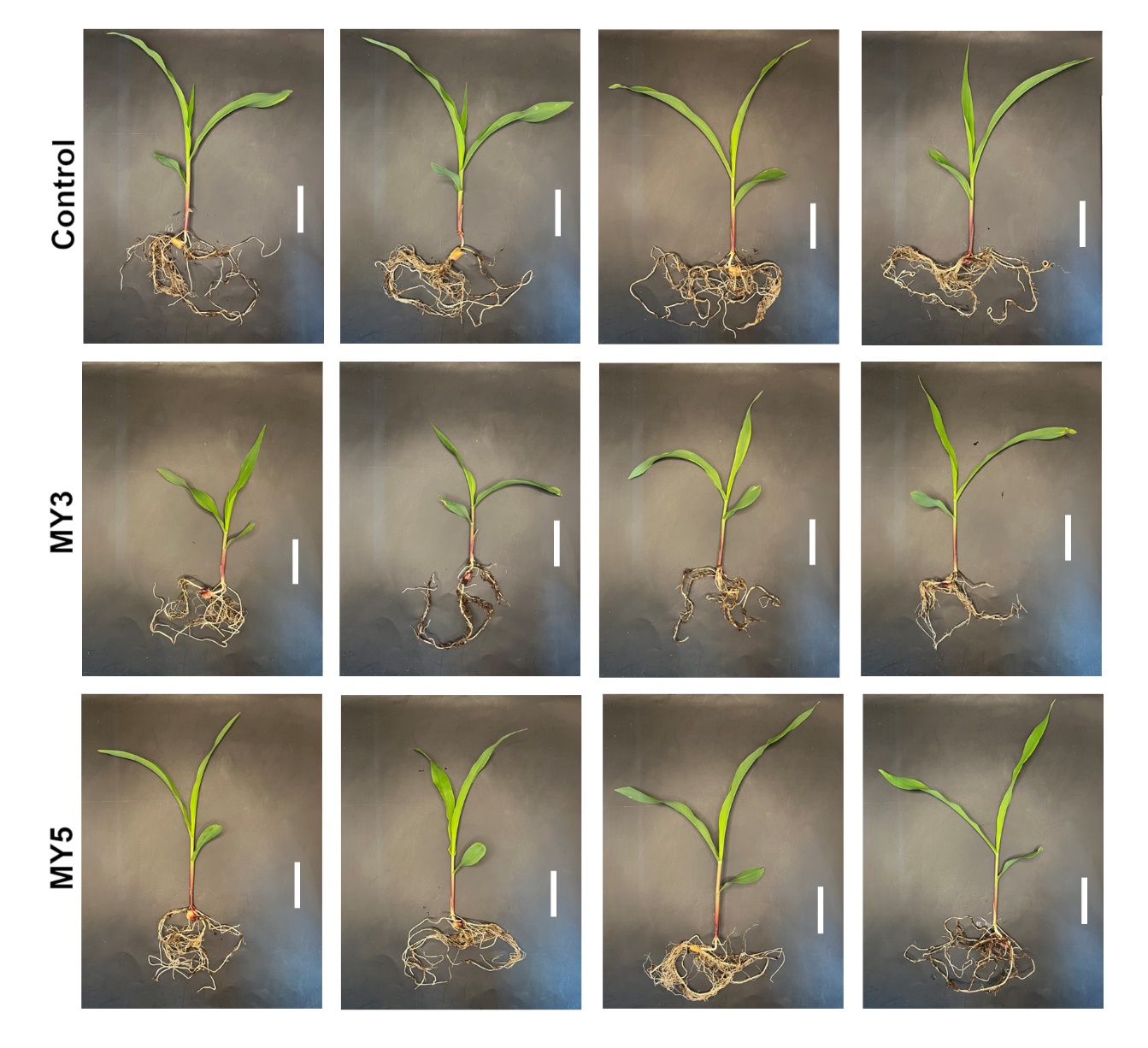


**Supplementary Figure 2. Effect of MY3 and MY5 *Fusarium verticillioides* strains in the growth of maize plants.** Maize seeds were infected with 9x10^3^ conidia/mL and then incubated in darkness for 3 days in Petri dishes and filter paper at 29°C. Seedlings were transplanted in plastic pots with Sphagnum Peat Moss turba (Premier Tech Horticulture. Quebec, Canada) and placed under 16/8 photoperiod for 15 days. Representative plants are shown. Bar= 5cm.
